# Incidence of Spontaneous Abortions During the COVID-19 Pandemic in a Regional County Hospital in Romania: A Retrospective Cohort Study

**DOI:** 10.3390/jcm14051418

**Published:** 2025-02-20

**Authors:** Diana Burlacu, Agnes Burlacu, Emmanuel Ladanyi, Bela Szabo, Tibor Mezei

**Affiliations:** 1 George Emil Palade University of Medicine, Pharmacy, Science, and Technology of Targu Mures, 540142 Targu Mures, Romania; agnesburlacu.endo@yahoo.com (A.B.); ladanyiemmanuel@gmail.com (E.L.); bela.szabo@umfst.ro (B.S.); 2 Department of Pathology, County Emergency Clinical Hospital of Targu Mures, 54000 Targu Mures, Romania; tibor.mezei@umfst.ro; 3 Department of Obstetrics and Gynecology, County Emergency Clinical Hospital of Targu Mures, 54000 Targu Mures, Romania

**Keywords:** pregnancy, miscarriage, stress, COVID-19 pandemic, viral infection

## Abstract

**Background**: The first trimester of pregnancy is known for its proinflammatory state, so it is considered a challenging period due to increased maternal vulnerability to viral infections. The main purpose of the current study was to evaluate the incidence trend of early miscarriages and whether there was any possible influence of the COVID-19 pandemic on pregnancy outcomes. **Materials and Methods**: We conducted a retrospective cohort study in which we included all pregnant women who had been admitted to our hospital between January 2018 and December 2022. Our aim was to compare the percentage of early miscarriages occurring in the pre-pandemic period (January 2018–February 2020) and during the pandemic (March 2020–December 2022). We decided to measure the total number and percentage of early pregnancy outcomes, including all viable pregnancies, ectopic pregnancies, and both medical and spontaneous abortions. **Results**: The annual incidence of registry-identified early miscarriages declined from 5.4% of 12–46-year-old women in 2018 to 3.6% in 2022 (*p* = 0.008). An overall incidence rate of 3.66% [95% C.I. 3.26–4.05] was calculated, with 4.25% [95% C.I. 3.35–4.41] in the pre-pandemic period and 3.24% [95% C.I. 2.82–3.57] during the pandemic. The highest incidence rate (*p* < 0.0001) was identified among nulliparous women (36.9%). **Conclusions**: To conclude, this study proved that the increase in the early miscarriage incidence rate could be assigned to an advanced maternal age, irrespective of one’s reproductive history. This study proved that no significant increase in the incidence rate of early miscarriage during the COVID-19 pandemic was noted, suggesting that this viral infection does not alter the risk of miscarriages. We hope that these findings help women deal with emotional stress and offer them reassurance about bearing children during pandemic periods.

## 1. Introduction

Spontaneous abortion or miscarriage is defined as the loss of a pregnancy before viability, with less than 20 weeks of gestation and a birthweight of less than 500 g. Approximately 23 million miscarriages are registered annually around the world [1].

Pregnant women are considered a vulnerable category to viral infections, especially during the first trimester, due to its well-known proinflammatory state [2]. Previous Coronavirus pandemics have been associated with adverse feto-maternal outcomes [3]. There are also other pandemics with a documented effect on pregnancy outcomes. A study published in 2016 provided evidence regarding adverse pregnancy outcomes in the case of the Zika virus pandemic. An increased incidence in the birth of newborns with microcephaly and maternal febrile rash illness was registered during 2015–2016. This may highlight the vertical transmission of the virus, but no linkage between Zika virus infection in pregnant women and abortion was encountered [4,5].

Prenatal diagnostic tests (chorionic villous sampling, amniocentesis, cordocentesis) primarily focus on the identification of genetic abnormalities in the fetus; however, they may also have a role in the identification of infectious diseases that may have an adverse effect on fetal development [6]. These tests aim to detect specific pathogens, including rubella, cytomegalovirus, toxoplasmosis, varicella, parvovirus, Zika virus, and many others, as these may cause the postpartum TORCH syndrome or other long-term complications [7,8].

The COVID-19 (Coronavirus disease) pandemic led to self-isolation during the first few months, causing fear, stress, and uncertainty regarding both the medical implications and unknown consequences deriving from this viral infection [9]. Although, at the present moment, there is a growing body of evidence regarding the impact of COVID-19 on maternal and perinatal outcomes, data on the real impact of the pandemic on the obstetric population in European countries still need to be made available.

The purpose of this study was to assess the possible adverse effects of the COVID-19 pandemic on early pregnancies in terms of miscarriage. Furthermore, it aimed to determine the change in the incidence of spontaneous abortions during the COVID-19 pandemic. We wanted to evaluate whether pregnancy outcomes had been influenced by epidemic-associated chronic stress.

## 2. Materials and Methods

Our retrospective cohort study included all pregnant women who had been admitted to the Obstetrics and Gynecology Clinic in the Targu-Mures Emergency Clinical Hospital, Romania, between 2018 and 2022. We compared the incidence of spontaneous abortions registered during the pre-pandemic and pandemic periods as follows: January 2018–February 2020 and March 2020–December 2022. The 11th of March 2020 was officially declared the beginning of the COVID-19 pandemic [10]. We decided to measure the total number and percentage of early pregnancy outcomes, including all viable pregnancies, ectopic pregnancies, and both medical and spontaneous abortions. We also categorized pregnant women into three groups: nulliparous women, women with a history of one viable delivery, and women with more than one viable delivery.

We defined early pregnancy as a pregnancy under 36 gestational weeks, after which we considered it an at-term pregnancy.

## 3. Results

Between 2018 and 2022, we identified 5624 women who had been admitted to the Obstetrics and Gynecology Clinic. Of these, 2326 had been admitted pre pandemic, while 3298 had been admitted during the pandemic.

**Figure 1:**
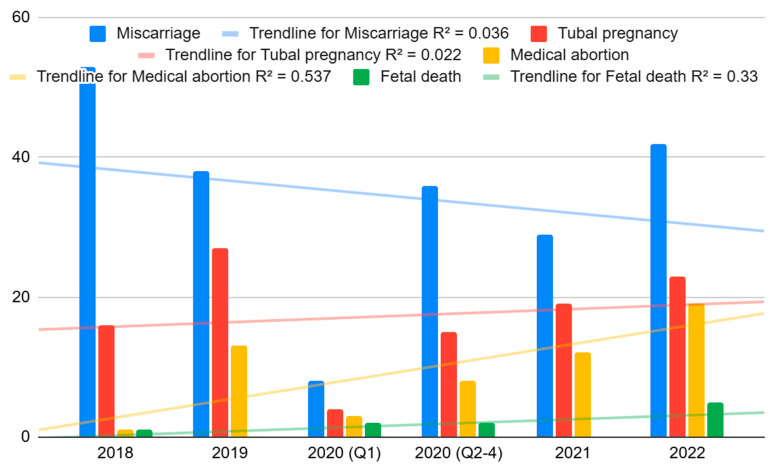
Trend of annual incidence rate of pathological pregnancies.

The annual incidence of registry-identified spontaneous abortions declined from 5.44% in 2018 to 3.57% in 2022. Moreover, the trend showed a significant decrease over these years (*p* = 0.008), with an R^2^ value of 0.036 (see Figure 1).

An overall incidence rate of 3.66% [95% CI 3.26–4.05] was calculated, with 4.25% [95% CI 3.35–4.41] in the pre-pandemic period and 3.24% [95% CI 2.82–3.57] during the pandemic.

Extreme maternal age groups also showed a decrease in the rate of spontaneous miscarriages. A total of 18.2% (pre-pandemic period) and 16.8% (pandemic period) of pregnant women suffered spontaneous abortions in the age group <20 years old, while 10.1% (pre-pandemic period) and 8.4% (pandemic period) women suffered spontaneous abortions in the age group >40 years old (*p* = 0.001).

**Figure 2:**
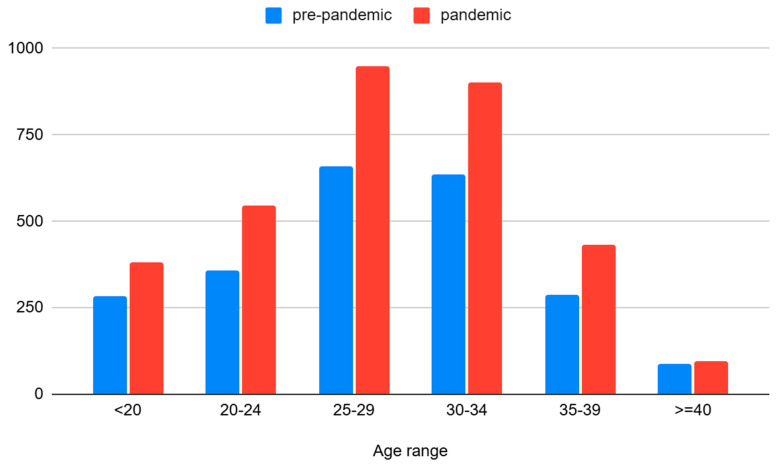
Miscarriages associated with maternal group age during the entire study period.

Maternal age was significantly associated with miscarriage (*p* = 0.018). In 2018, pregnant women aged 25–29 years had the highest percentage of miscarriages (29.9%). During 2019, 2020, 2021, and 2022, the highest percentage of miscarriages occurred in the maternal group 30–34 years old: 28.9% (2019), 27.9% (2020), 31.1% (2021), and 28.4% (2022) (see Figure 1 and Figure 2).

**Figure 3:**
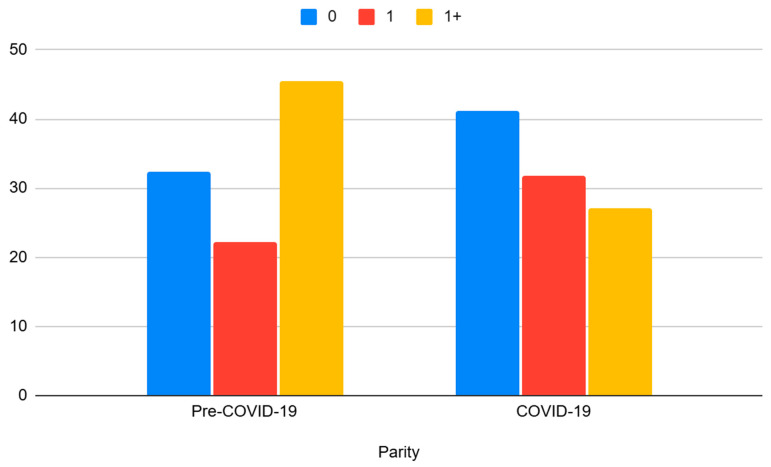
Comparison of miscarriage trend correlated with women’s parity between the two study periods.

Our data indicated a higher incidence rate of miscarriages occurring among nulliparous women, compared to parous women, after categorizing all pregnant women in this study into the three groups. Hence, the proportion of all miscarriages among registry-identified pregnancies during the first study period was as follows: 32.3% were attributed to nulliparous women, 22.2% to primiparous women with a history of one viable delivery, and 45.5% to multiparous women with a history of more than one viable delivery. During the second study period, these proportions increased to 41.1% among nulliparous women (*p* < 0.001) and 31.8% among primiparous women with a history of one viable delivery, while they decreased to 27.1% among multiparous women with a history of more than one viable delivery (*p* < 0.001) (see Figure 3).

During 2018–2022, a total of 20.4% registry-identified early pregnancies were noted, with 18.9% in 2018, 23.5% in 2019, 19.8% in 2020, 18.0% in 2021, and 21.8% in 2022 (*p* = 0.012). A total of 19.5% and 20.1% of early pregnancies occurred during the pre-pandemic and pandemic periods, respectively.

During the pandemic period, we observed a significant association between parity and gestational age at the time of delivery, with 30.9% of nulliparous women, 20.1% of primiparous women, and 19.5% of multiparous women having early pregnancies (*p* = 0.044).

**Figure 4:**
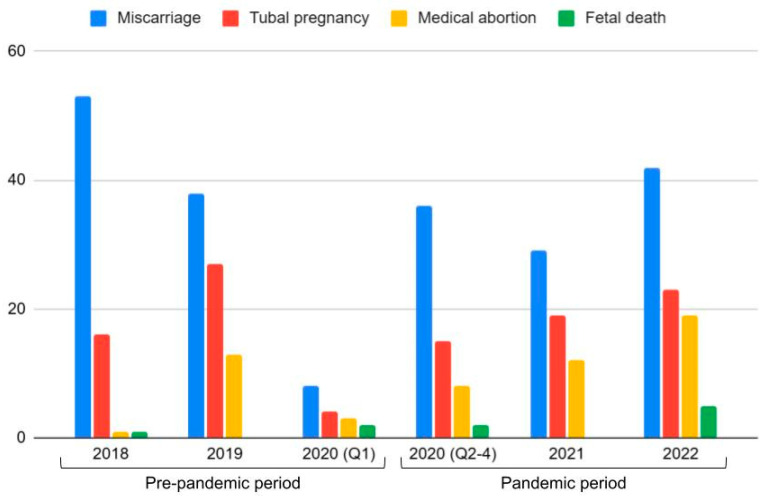
Incidence of pathological pregnancies before and during the pandemic.

Of all registered pregnancies during the study interval, 377 (6.7% of all pregnancies) were considered pathological. These included ectopic pregnancies (104, 1.84%), medically justified abortions (57, 1.01%), spontaneous abortions (206, 3.66%), and fetal demise (10, 0.19%) (*p* = 0.002), as shown in Figure 4.

During the pandemic, no statistical association was noted between spontaneous abortions and SARS-CoV-2 active infection (*p* = 0.978). Only 0.9% of spontaneous abortions and one maternal death were associated with viral infection.

## 4. Discussion

Miscarriage has been intensively studied, since it is among the commonest adverse pregnancy outcomes. More than 15% of clinically recognized pregnancies may have this outcome [11,12]. As some recently published data indicate, our study demonstrated a higher incidence of miscarriages with an increasing maternal age, specifically in women aged 30 years or more [13,14].

Two register-based studies presented evidence regarding the highest risk of miscarriage in women aged 45 years or more, proving that the rate of miscarriage increased with women’s parity [15]. In contrast, we noted that the biggest proportion of all registry-identified miscarriages was mostly seen among nulliparous women. When comparing nulliparous and parous women, more than half of all registry-identified miscarriages occurred in nulliparous women.

It has been previously theorized that women facing miscarriages lack certain factors that would lead to full-term pregnancy development and not recurrent spontaneous abortions. Several hypotheses have been proposed to support such an “absent factor” mechanism. In previous studies, an IG antibody that could lead to total blockage of the mixed lymphocyte reaction was thought to be either absent or present in low titers in samples from women with recurrent spontaneous abortions [16,17].

The more HLA antigens are shared between partners, the higher the risk of spontaneous abortion. Consequently, women who share a variable amount of HLA types with their partners might experience an adverse pregnancy outcome, depending on the segregation of their genotype [18,19]. Still, these immunological theories were not fully reinforced by the clinical studies that followed [20,21].

On the other hand, another theory proposed by Clarke et al. postulated that any residual material in the uterine cavity after a miscarriage might jeopardize future pregnancies. According to this hypothesis, any possible “rest” from trophoblastic cells would influence the outcome of a pregnancy, since anencephaly or spina bifida were documented in women whose last pregnancy ended in miscarriage compared to controls whose outcome was a typical baby [18,22]. Prospective studies are needed to identify which factors could determine whether a first pregnancy will result in success or failure, thus offering prophylactic pre-conceptual counseling to nulligravid women.

In many instances, however, the exact cause of a miscarriage remains unknown. Multiple risk factors are considered, such as maternal age, genetics, and hormonal and environmental factors. A recent study suggested that parental chromosomal rearrangements could determine more than 50% of all recurrent miscarriages [23]. An advanced maternal age has been found to be one of the most significant risk factors. Consequently, in approximately half of spontaneous abortions before a gestational age of 12 weeks, fetal chromosomal abnormalities due to meiotic errors are found, caused by an advanced maternal age at the time of conception [15,24,25]. Another study found that autosomal trisomies are the most common genetic anomalies (52% of all miscarriages), followed by polyploidy (21%) and, finally, monosomy X (13% of cases) [26].

Although common, affecting one in ten women throughout their lifetime, miscarriage also has common risk factors, such as increasing age in both partners. In our study, this risk factor was also highlighted as being related to miscarriage. Furthermore, permanent stress as an adjustment to the lifestyle that modern life entails, along with daily habits (alcohol consumption and cigarette smoking included), outlines the typical portrait of a woman who is more prone to suffer a miscarriage [26]. In the general population, patients diagnosed with COVID-19 were more susceptible to developing thromboembolism due to excessive inflammation, hypoxia, and immobilization [26].

This misconception, postulating that miscarriages are often inevitable and that a woman who has suffered such a loss should keep trying to become pregnant until, eventually, she is able to carry at least one pregnancy to term, should belong to the past. This thinking can lead to psycho-emotional damage for the families in question. Regarding the possibility of a subsequent successful pregnancy, miscarriage is often associated with several fetal risk factors, namely preterm birth and fetal growth restriction [26]. This supposed increase in preterm delivery risk has been preliminary reported as varying up to 47%. Although insufficient, the correlation between COVID-19 infection, miscarriage, and a subsequent preterm delivery has been suggested based on some reported cases due to the premature rupture of membranes [26].

Moreover, spontaneous abortion can be a significant trigger for depression and anxiety in women, representing a significant psychological burden for them [27]. Mostly, they may struggle with feelings of guilt over what they have done wrong or what they could have done differently to prevent the loss of the pregnancy. Many studies have discovered an impressive percentage of women prone to psychiatric symptoms in the weeks to months after a pregnancy loss, most of them not having children [28,29]. The majority of women suffering a miscarriage are willing to know whether there is anything they can do to prevent a future miscarriage. These couples usually seek health care advice on how long they should be waiting before trying to conceive again [30]. Still, a consensus on an appropriate interpregnancy interval after suffering a pregnancy loss is lacking. The World Health Organization (WHO) only recommends waiting at least six months, this limitation being based on a single cross-sectional study [31,32].

The beginning of the COVID-19 pandemic caused the self-isolation of community members, leading to stress, fear, and economic instability, alongside other already known factors. The chronic stress associated with this situation might have had a significant influence on pregnancy outcomes. A systematic review from 2017 suggested this hypothesis when studying the exposure of pregnant women to armed conflict [33]. Another study addressed a possible linkage between pregnancy outcomes and exposure to stress caused by rocket-attack alarms, showing a high risk of spontaneous abortion [34].

The Middle East Respiratory Syndrome (MERS) and Severe Acute Respiratory Syndrome (SARS) pandemics have both been studied regarding their possible impact on adverse pregnancy outcomes. Indeed, these two pandemics have been associated with increased maternal morbidity and mortality. Despite the small number of reported cases confirmed with MERS and SARS infection during pregnancy, the data revealed a feto-maternal fatality rate of almost 30% [30].

Infection with SARS-CoV during the 2002–2003 epidemic determined more than 50% of spontaneous abortions. During this pandemic from the early 2000s, an increased fatality rate was studied among pregnant patients. With a fatality rate of 25%, this outcome could be attributed to several physiological changes in pulmonary function usually occurring during advanced pregnancy [35].

No vertical transmission of the virus was detected, as no viral particles were discovered in the products of conception or after the histopathological examination of the placenta. Instead, the main causes of miscarriages during this pandemic period were severe maternal respiratory failure and hypoxemia. A more comprehensive description was published after the placentas of patients infected with SARS-CoV were examined: multiple cases of placental infection suggesting maternal vascular malperfusion (MVM) lesions and avascular villi suggesting fetal thrombosis were reported, alongside numerous cases of significant placental weight reduction. These histopathological changes were more likely related to hypoxemia and feto-maternal circulatory insufficiency as a direct result of SARS-CoV infection. Furthermore, in other studies, severe adverse pregnancy outcomes have also been associated with Middle East Respiratory Syndrome Coronavirus (MERS-CoV) [36,37].

There is growing evidence related to COVID-19’s association with miscarriage [38,39,40]. Pregnant women belong to a category of people known to be at risk for numerous comorbidities. Although no increased risk related to COVID-19 infection was encountered among pregnant women compared to the remaining population in Ref. [40], almost 85% of pregnant women were thought to experience mild COVID-19 infection symptoms, 10% more severe disease, and 5% critical disease. The most commonly reported symptoms were shortness of breath, fever, and dry cough [40].

Vertical maternal–fetal transmission has been previously documented when maternal infection with TORCH syndrome and Zika virus was serologically confirmed, causing important fetal consequences. The first trimester of pregnancy has a critical importance, since fetal organs develop during this time. Hence, any maternal infection at this point could have an adverse impact on fetal development, compared to later gestational weeks [36,37].

Vertical transmission of this viral infection can occur during fetal intrauterine life through the placenta, during delivery through the aspiration of cervical–vaginal secretions, and through breastfeeding postpartum [40]. No evidence of transplacental transmission of COVID-19 exists [41], although several observational studies regarding this possibility have been published [40].

No significant increase in the risk of miscarriage in patients infected with COVID-19 during the first trimester has been observed [3,42]. Rather, studies related to miscarriage in pregnant women bearing this infection have shown that most miscarriages occur due to placental insufficiency, preterm delivery, and intrauterine growth restriction [37,43].

Solid evidence regarding the effect of COVID-19 infection on placental pathology is still limited, but given the amount of vascular maternal adverse outcomes, more frequent maternal vascular malperfusion (MVM) and fetal vascular malperfusion (FVM) have been observed [40]. On the other hand, many studies reported so far have revealed the absence of any particular connection regarding COVID-19 infection and most placental pathologies, when compared with controls [44,45,46].

The maternal organism’s homeostasis has a physiological reaction to stress, with hypothalamic–pituitary–adrenal (HPA) axis activation as the first mechanism. The activation of this axis determines the release of the corticotropin-releasing hormone (CRH), which activates the adrenocorticotropic hormone, eventually leading to cortisol release from the adrenal gland. The latter, referred to as the stress hormone, then negatively impacts physiological mechanisms, thus becoming unable to properly restore homeostasis [47].

The literature reveals numerous studies that have determined a linkage between adverse pregnancy outcomes and levels of cortisol [48]. Lower birth weight, prematurity, and miscarriage have also been encountered in said studies. It is worth mentioning that not only high levels of cortisol have been discovered to be associated with adverse outcomes, but also a decrease in cortisol levels [49]. The vasoconstriction caused by a high level of norepinephrine has been found to be related to adverse pregnancy outcomes, since it may impair the uterine arteries, which could lead to inappropriate placental perfusion.

Angiotensin-converting enzyme 2 (ACE2) has been studied as the cellular entry receptor for SARS-CoV-2. During pregnancy, the feto-maternal interface is represented by both the placenta and the decidua. Hence, viral receptor expression at the level of the placenta or decidua represents the viral infection promoter. In a previous study of a placenta at 14 gestational weeks, four main cell types were shown to reveal ACE2 expression during single-cell transcriptome genomic data sequencing: stromal cells, villous cytotrophoblast, and syncytiotrophoblasts from the placenta and perivascular cells from the decidua. A high expression of ACE2 at the level of these cells implies adverse feto-maternal outcomes, given the possibility of placental dysfunction, insufficiency, and subsequent pregnancy complications [40].

At the present moment, there are few publications on the outcomes of pregnant women with COVID-19, most of them related to women in the second or third trimester [42]. Many viral infections can be harmful to the fetus during the first trimester of pregnancy, and whether Severe Acute Sars-Cov2 is one of them is yet to be determined. Data on COVID-19 during pregnancy remain limited, mostly acquired from limited sample studies [44,45,46].

During the pandemic, both positive and negative aspects were experienced by pregnant patients and their families. As expected, a sense of loss of the birthing experience under normal circumstances was felt, since the hospital policies limited family members’ visits. Panic and overall emotional stress were encountered upon positive Sar-Cov-2 testing. On the other hand, such strict policies led to a more profound and intimate feto-maternal connection, without the presence of other family members [47,48,49].

Our study had some limitations. Firstly, this was a single-center study that used registry data to identify miscarriages. We only had access to information for miscarriages diagnosed and treated within the public specialized health care sector. Thus, women with early pregnancy loss who possibly never sought medical attention were likely not included in this study.

It is also worthwhile to note that the pandemic could have potentially changed women’s willingness to visit hospital environments regularly. As such, pregnant women might have had doubts about their safety in the hospital environment and, some of them, might have decided to put an end to their prenatal medical visit routine, presenting to the hospital only when the viability of the pregnancy could no longer be helped.

The retrospective nature of this study prevented us from using tools to compare stress levels between women who had experienced miscarriages and those who did not. However, we believe that the pandemic had an impact on almost every aspect of people’s lives, serving as a unique stressor.

Early pregnancy was not exempt from such stress, and our study aimed to explore some of the effects of this situation. The full impact of the recent pandemic on reproduction is a complex issue, and many aspects are yet to be determined.

## 5. Conclusions

To conclude, this study proved that the observed increase in the early miscarriage incidence rate could be assigned to an advanced maternal age, irrespective of patients’ reproductive history. This study proved that no significant increase in the incidence rate of early miscarriage during the COVID-19 pandemic was noted, suggesting that this viral infection did not alter the risk of miscarriages. Meanwhile, the increased average age of primiparous women could have been another reason for the higher proportion of miscarriages among nulliparous women.

We hope that these findings help women deal with emotional stress and offer reassurance about bearing children during pandemic periods. The present study is the first Eastern European study to investigate the overall declining incidence rate of early miscarriage during the COVID-19 pandemic.

## Data Availability

Data available on reasonable request due to ethical restrictions.
